# Do the Memory Support Intervention and Improving Memory for Treatment Facilitate Behavior Change in Cognitive Therapy?

**DOI:** 10.1007/s10608-025-10629-7

**Published:** 2025-06-18

**Authors:** Courtney Armstrong, Nicole Gumport, Allison Harvey

**Affiliations:** 1 University of California, Berkeley, Berkeley, USA; 2 Stanford University, Stanford, USA

**Keywords:** Behavior change, Cognitive therapy, Theoretical Domains Framework, Memory for treatment

## Abstract

**Background:**

The memory support intervention (MSI) was developed to improve patient memory for treatment as poor memory for treatment is associated with poorer adherence and outcomes. This study aimed to (1) validate the Cognitive Behavior Change interview, which was developed for and used as a measure in this study, (2) assess differences in specific factors impacting behavior change (domains) described in the Theoretical Domains Framework (TDF) among participants who received the MSI alongside cognitive therapy (CT) compared to those who received CT-as-usual, and (3) assess whether memory for treatment facilitates behavior change.

**Methods:**

Participants (*N* = 64) from a trial of adults with Major Depressive Disorder were randomly allocated to CT + Memory Support (MS) or CT-as-usual. To ascertain whether CT + MS better improves knowledge of domains of behavior change, the Cognitive Behavior Change Interview was administered at the 12-month follow-up (12FU) assessment. This qualitative and quantitative interview is grounded in the TDF and includes ratings of potential facilitators of behavior change. Regression was used to examine relations between memory support, these facilitators, and behavior change.

**Results:**

Internal consistency for the Cognitive Behavioral Change interview fell in the acceptable range, whereas interrater reliability ranged from unreliable to excellent. Participants who received CT + MS demonstrated better overall engagement with domains compared to participants who received CT-as-usual. Improved memory for treatment was positively associated with overall engagement and the number of domains engaged. Overall engagement and the number of domains engaged were associated with utilization of skills learned in CT and improved impairment and depression at 12FU.

**Conclusions:**

This study enhances our understanding of the potential role of memory for treatment improving long-term behavior change. Exploratory analyses highlight specific domains that may be factors driving changes in behavior due to CT resulting in improvements in depression.

**Supplementary Information:**

The online version contains supplementary material available at 10.1007/s10608-025-10629-7.

## Introduction

Adherence to evidence-based psychological treatments (EBPT) is associated with improved outcomes related to mood, sleep, and psychiatric symptoms (Decker et al., 2016; Dong et al., 2017; LeBeau et al., 2013). Such adherence almost always involves behavior change (DiMatteo et al., 2012). Despite growing awareness of the benefits of behavior change, recipients of EBPTs regularly struggle to initiate behavior change over the course of treatment (Wood & Neal, 2016). In cases where change occurs, maintaining changes in the long-term remains difficult, and relapse into unhealthy behaviors is common (Wood & Neal, 2016), with negative consequences for overall clinical improvement. As such, it is important that EBPTs utilize techniques that facilitate behavior change and maximize a recipient’s ability to sustain change over time (Sumner et al., 2018).

Improving memory for the contents of treatment may be one method to facilitate behavior change. Memory for treatment is generally poor (Bober et al., 2007; Flocke & Stange, 2004): the average patient recalls fewer than half of the recommendations made during a treatment (Flocke & Stange, 2004) and facts are often remembered incorrectly (Bartlett et al., 1984; Bober et al., 2007). Recall is particularly poor for health behavior change advice (Flocke & Stange, 2004; Kravitz et al., 1993), which can negatively affect treatment adherence (Sarfan et al., 2022; Wiegand et al., 2013). Given these findings, it is important to investigate whether improving memory for treatment facilitates behavior change and maintenance of behavior change—and ultimately, clinical outcomes.

The memory support intervention (MSI) was developed to improve patient memory for treatment by adding eight memory support strategies, derived from the cognitive science and educational literature, to treatment-as-usual (Harvey et al., 2017). A pilot trial demonstrated the potential efficacy of the MSI (Harvey et al., 2016), and evidence exists that improved memory for treatment positively affects patient adherence in adults with major depressive disorder (MDD) (Dong et al., 2017). More recently, as part of a large, fully-powered confirmatory randomized controlled trial (RCT)—henceforth referred to as the “parent trial”—the MSI was delivered alongside cognitive therapy (CT) to adults with MDD (Dong et al., 2022).

The MSI was designed to be transdiagnostic (i.e. relevant across disorders) and pan-treatment (i.e. to work in conjunction with multiple treatment types). CT for depression was selected as the focus for the parent trial because MDD is prevalent and causes impairment (Kessler et al., 2003)—and CT is an evidence-based psychotherapeutic treatment for MDD (Spinhoven et al., 2018; Strunk & DeRubeis, 2001). However, a substantial portion of MDD patients do not respond optimally to CT, and a portion of those who do improve eventually relapse (Bockting et al., 2015). Thus, there is room for improvement in CT for MDD. Poor memory for treatment has been shown to be common and modifiable in MDD (Taconnat et al., 2010). As such, it is reasonable to believe that individuals with MDD stand to benefit from adding memory support for the content of treatment.

The present study seeks to explore the relation between the MSI and cognitive and behavioral change through a theoretical lens. Theories of behavior change have a vital role to play in the design and evaluation of EBPTs. Indeed, incorporating theories of behavior change into the design and evaluation of EBPTs has been shown to lead to increased efficacy and improved outcomes (Kok et al., 2016). Additionally, behavior change theory can help researchers identify factors that influence patient behavior and promote a better understanding of mechanisms of change within an intervention (Michie, 2008). Specifically, the Theoretical Domains Framework (TDF) will be used. Created by 87 behavior change experts, the TDF draws from 33 behavior change theories and 128 explanatory constructs to present 14 basic, frequently occurring domains (Cane et al., 2012). Table 1 presents definitions of the 14 TDF domains as presented in Cane et al. (2012). All domains are listed and defined in Table 1. The TDF does not propose testable relations between domains but instead highlights known influences on cognitive and behavioral change that are common across multiple theories, allowing for a larger range of potential explanations for behavior than what may be provided by a single theory. Thus, this framework is ideal for the current study, which seeks to explore facilitators of behavior change among participants who received memory support compared to those who did not.

**Table 1:** Definitions of domains within the theoretical domains framework

Domain	Definition^a^
Knowledge	An awareness of the definition of something
Skills	An ability or proficiency acquired through practice
Social/professional role and identity	A coherent set of behaviors and displayed personal qualities of an individual in a social or work setting
Beliefs about capabilities	Acceptance of the truth, reality, or validity about an ability, talent, or facility that a person can put to constructive use
Optimism	The confidence that things will happen for the best or that desired goals will be attained
Beliefs about consequences	Acceptance of the truth, reality, or validity about outcomes of a behavior in a given situation
Reinforcement	Increasing the probability of a response by arranging a dependent relationship, or contingency, between the response and a given stimulus
Intentions	A conscious decision to perform a behavior or a resolve to act in a certain way
Goals	Mental representations of outcomes or end states that an individual wants to achieve
Memory, attention and decision processes	The ability to retain information, focus selectively on aspects of the environment and choose between two or more alternatives
Environmental context and resources	Any circumstance of a person's situation or environment that discourages or encourages the development of skills and abilities, independence, social competence, and adaptive behavior
Social influences	Those interpersonal processes that can cause individuals to change their thoughts, feelings, or behaviors
Emotion	A complex reaction pattern, involving experiential, behavioral, and physiological elements, by which the individual attempts to deal with a personally significant matter or event
Behavioral regulation	Anything aimed at managing or changing objectively observed or measured actions

^a^All definitions are based on definitions from the American Psychological Associations’ Dictionary of Psychology (2007), as presented in Cane et al (2012)

The TDF was initially conceptualized and designed to help implementation researchers identify influences on health-professionals’ behavior, specifically related to the implementation of evidence-based recommendations (Atkins et al., 2017). It has since been extended to other areas, such as designing behavioral interventions, explaining intervention outcomes (Curran et al., 2013; Newlands et al., 2021), changing patient health behaviors (Nicholson et al., 2014), and changing general population behaviors (Honigh-de Vlaming et al., 2013). As such, the TDF is useful for informing multiple stages of intervention design and implementation. The TDF also has served as the basis for numerous qualitative interviews and informed the design of several survey instruments. Many of these measures demonstrate the TDF’s utility in identifying determinants of behavior and behavior change (Curran et al., 2013; Faija et al., 2020; Huijg et al., 2014). Accordingly, the TDF was used as the basis for this research.

The present study sought to improve our understanding of the role that memory support plays in behavioral and cognitive change through the development and administration of a semi-structured, mixed methods interview. This interview, called the Cognitive Behavior Change Interview, is presented in Appendix 1. It involves both qualitative and quantitative components, constructed for this study, and is grounded in the TDF (Atkins et al., 2017; Cane et al., 2012). The purpose of the interview was to fully explore each domain of the TDF within the context of CT. Each question was designed to investigate or assess a specific TDF domain related to the management and reappraisal of negative thoughts. This area was selected as the focus of the interview, as it is a key component of CT that all participants receive. Knowledge of whether the MSI engages the TDF domains will provide information on how memory support facilitates change in evidence-based psychotherapies.

This study addresses the following aims through the development and administration of a theory-based, semi-structured mixed-methods interview—the Cognitive Behavior Change Interview—which was delivered to participants in the parent trial at the 12-month follow-up interview. Considering that the Cognitive Behavior Change Interview was developed for this research study, it will be important to evaluate the measure. The 12-month follow-up was selected as the time point because behavior change is difficult to sustain over time. Thus, it is important to understand which TDF domains continue to influence behavior well after the conclusion of treatment, which included 4 months of CT (20–26 sessions of therapy).

The first aim is to evaluate the internal consistency and interrater reliability of the Cognitive Behavior Change Interview. It was hypothesized that the Cognitive Behavior Change Interview will have acceptable internal consistency and interrater reliability. The second aim was to use the Cognitive Behavior Change Interview to understand how the MSI and memory for treatment impact behavior change domains. This aim has two parts. First, to establish whether participants who received CT + Memory Support (CT + MS) compared to CT-as-usual differ on Overall Engagement, Total Domains, or individual domain scores. The hypothesis tested is that Overall Engagement, Total Domains, and individual domain ratings will be higher in CT + MS, compared to CT-as-usual. Second, to examine whether patients who exhibit better memory for treatment at the post-treatment assessment score higher on Overall Engagement, Total Domains, and individual domains at the 12-month follow-up. It was hypothesized that participants with better memory for treatment will score higher on Overall Engagement, Total Domains, and Individual Domains. Memory for treatment was assessed at the post-treatment timepoint to minimize the amount of time participants had to forget treatment points, and to establish temporality. The third aim was to determine the relation between (a) Overall Engagement, Total Domains, individual domains, and (b) selected treatment outcomes (treatment utilization, disability, and depression). It was hypothesized that Overall Engagement, Total Domains, and individual domains would positively predict better treatment outcomes.

## Methods

### Participants

This study included 64 consecutively randomized participants who met diagnostic criteria for MDD and received CT as part of the NIMH-funded parent trial (Dong et al., 2022; Harvey et al., 2017; Zieve et al., 2022). This study once the parent trial was underway. Thus, the participants were people from the parent study who had yet to complete their 12-month follow-up visit. For the parent study, participants were recruited via advertisements and clinician referrals. Eligibility was initially assessed via phone interview, and then a more detailed in-person interview.

Inclusion and exclusion criteria for the parent trial were selected to enhance representativeness and generalizability and thus were kept to a minimum. The inclusion criteria for the parent trial were: (1) age 18 + years old; (2) willing and able to consent to being video-recorded and to NIMH data sharing; (3) English language fluency; (4) diagnosis of major depressive disorder (MDD), first episode, recurrent or chronic according to the *Diagnostic and Statistical Manual of Mental Disorders, Fifth Edition* (DSM-5); and (5) minimum score of 26 or above on the Inventory of Depressive Symptomatology, Self-Report (IDS-SR)—a cutoff denoting at least “moderate” depression; and 6) if taking medications for mood, medications must have been stable for the past four weeks.

### Treatments

Both treatment conditions involved 16 weeks (20–26 sessions) of CT for depression. Treatment was administered by a licensed therapist or graduate student in clinical psychology or social work.

#### CT-as-Usual

CT was developed by Beck (1979). Treatment maneuvers are designed to identify, reality test, and correct distorted beliefs and information processing. CT for MDD was conducted according to the standard manuals (Beck, 1979).

#### CT + MS

The MSI is a manualized adjunctive treatment that was delivered alongside CT-as-usual. Taken from the cognitive science and education literature based on carefully honed criteria (Harvey et al., 2014), the MSI is composed of eight memory support (MS) strategies: attention recruitment, categorization, evaluation, application, repetition, practice remembering, cue-based reminder and praise recall. Definitions as described in previous research (Harvey et al., 2014) are provided in Table 2. These strategies are proactively, strategically, and intensively integrated into treatment-as-usual to support encoding. Memory support is delivered alongside each “treatment point,” defined as a main idea, principle, or experience that the treatment provider wants the patient to remember or implement as part of the treatment (Lee & Harvey, 2015).

**Table 2:** Memory support strategies with definitions

Strategy	Definition
Attention recruitment	Directing attention to specific therapy points via emphasis or scaffolding
Categorization	Organizing therapy points into distinct classification
Evaluation	Reviewing the pros and cons of using a skill or adopting a belief
Application	Prompting the participant to describe how a treatment may be relevant to their individual life
Repetition	Repeating treatment points for emphasis
Practice remembering	Asking participants questions that prompt recall of treatment points
Cue-based reminder	Preemptively establishing cues to help participant remember to use a skill or consider a treatment point
Praise recall	Reinforcing correct recall of treatment points

Some memory support is a standard part of certain treatments, including CT. However, providers often deliver a sub-optimal “dose” of memory support that does not improve memory for treatment (Lee et al., 2020). The MSI is designed to ensure that an adequate dose of memory support is provided. Previous research suggests that increasing the dose of memory support via the MSI can improve depression symptoms (Harvey et al., 2016).

### Measures

#### Patient Recall Task

The Patient Recall Task (Lee & Harvey, 2015) is a free recall measure of patient memory for cognitive therapy and was used to measure patient memory for treatment. Over the course of the parent trial, participants regardless of treatment condition were given a sheet of paper and asked to recall as many points from treatment as they could remember. The total number of points remembered served as their cumulative recall. Coders then determined the overall number of distinct treatment points recalled via the scoring rubric. The Patient Recall Task has strong interrater reliability, strong predictive validity of future memory for treatment (*r*'s = 0.29–0.36, *p* = 0.022–0.073), and is associated with the amount of memory support delivered (Lee & Harvey, 2015). 

#### The Cognitive Behavior Change Interview

With the goal of covering the full range of ways that changes in behavior and cognition may be facilitated, an in-depth, a one-on-one interview design was deemed to be most appropriate (Curry et al., 2009). The Cognitive Behavior Change Interview was developed by the research team to address this goal. It is a semi-structured mixed-methods interview composed of both qualitative and quantitative components. It consists of seventeen questions and was designed for this study to explore the role of all 14 domains highlighted in the TDF to facilitate behavior change. Questions were designed based on past research and studies using the TDF (Nicholson et al., 2014), and constructs that defined a given domain (Cane et al., 2012). At least one question was asked for each TDF domain. One additional question was developed for each of three domains (Memory Attention and Decision Processes, Optimism, and Beliefs about Capabilities) because these domains embodied a wider range of constructs compared to other domains. As such, one additional question per domain was developed to ensure all vital constructs were properly represented within the interview. The interview questions examine how each domain may influence the adoption of skills and techniques learned in CT for the purpose of managing negative thoughts. The management of negative thoughts was selected as the focus because the management of negative thoughts via skill such as cognitive restructuring is a central component of CT. The Cognitive Behavior Change Interview is included in Appendix 1.

#### Utilization Questionnaire

The utilization of skills learned in treatment was measured using the total score from the Utilization Questionnaire (Gumport et al., 2019). This measure is the index of the actual behavioral changes made by participants. Collected during the 12-month follow-up, this questionnaire lists 14 core skills used in CT. Each skill is rated by the participant according to how frequently the skill is utilized. Each skill is rated on a 5-point Likert scale (0 = *I never use it*; 1 = *I rarely use it*; 2 = *I occasionally use it*; 3 = *I often use it*; 4 = *I always use it*). Cronbach’s alpha for this scale is 0.84, which is considered good (Gumport et al., 2019, 2023). Total Utilization scores were created by taking the mean of all items on the scale (Gumport et al., 2019). Utilization was selected as an outcome of interest for this study because the utilization of skills learned in treatment is a direct measure of behavior.

#### World Health Organization Disability Assessment Schedule (WHODAS)

The WHODAS measured functional impairment. Collected at the 12-month follow-up, this 36-item, self-administered questionnaire assesses functioning in everyday situations over the last 30 days. Functioning is measured across six domains: (1) cognition, (2) mobility, (3) self-care (such as hygiene, dressing and eating), (4) getting along with others, (5) life activities (the ability to respond to everyday responsibilities), and (6) participation in society. The WHODAS has excellent psychometric properties (Üstün et al., 2010). Scores range from 0 to 144 with higher scores indicating higher levels of impairment (i.e., lower levels of functioning).

#### The Inventory of Depressive Symptomatology Self Report (IDS-SR)

The IDS-SR is a 30-item questionnaire measuring depressive symptoms collected at the 12-month follow-up. Each item consists of four statements that describe various degrees of severity regarding specific symptoms of depression. These items are scored on a four-point scale ranging from 0 to 3. Total scores range from 0 to 84, with higher scores indicating more severe depression (Rush et al., 1996). This measure was selected as an outcome because reduction in depression symptoms is a primary outcome of the parent trial.

#### Demographics

A demographics form collected at baseline assessed patient demographics including years of education. As years of education has been shown to impact the effects of memory support (Harvey et al., 2016), it was included as a covariate across models.

### Procedure

All procedures were in accordance with institutional and national standards aligned with principles from the Declaration of Helsinki. All procedures were approved by the University of California, Berkeley Institutional Review Board. All participants provided informed consent.

Participants in the parent trial completed 16 weeks and a minimum of 20 sessions of either CT-as-usual or CT + MS. A maximum of 26 sessions of treatment was provided. Following the completion of treatment, participants completed assessments at post-treatment, 6-month follow-up, and 12-month follow-up. The present research was completed during the 12-month follow-up assessment. The 12-month follow-up assessment was selected because behavior change is difficult to sustain over time. Thus, observing differences between participants with and without the MSI 12 months after CT has concluded is of considerable interest.

The Cognitive Behavior Change Interview was conducted in-person by one of two trained interviewers. The duration of the interview ranged from 25 to 35 min. During the interview, the interviewer asked the participant about their ability to identify and restructure negative thoughts, and how behavioral domains listed in the TDF may facilitate a skill used to manage negative thoughts. In circumstances where a participant gave a vague or uninformative answer, interviewers used pre-written prompts to elicit further detail or information from the participant without leading the participant towards desired responses. Recordings of the interview were then shared with trained coders, who noted any deviation from the interview guide, missed opportunities to probe, or missed questions before coding the recorded interview.

### Data Scoring and Analyses

#### Coding of the Cognitive Behavior Change Interview

Coding the Cognitive Behavior Change Interview relied on assigning global ratings reflecting the extent to which the behavior of the participant was facilitated by a given domain within the TDF. This approach follows the procedures used in prior research (Collins & Feeney, 2000; Van Vleet et al., 2018). This approach is considered to be a form of “Quantitizing,” a common component of mixed-methods research in which qualitative data are numerically translated and made fit for quantitative analysis (Sandelowski et al., 2009). This is done so that data collected qualitatively may still be used to test hypotheses and address relations between variables while minimizing the loss of nuance (Sandelowski et al., 2009). Overall Engagement was determined by taking the average across all domain ratings.

Each Cognitive Behavior Change Interview was coded by two treatment-blind, trained independent coders for the domains that comprise the TDF. To ensure a rigorous coding process, coders listened to the entire recording of the interview at least twice: once to become familiar with the interview in its entirety, and then a second time stopping frequently to take notes on the presence of a given domain. Coders were instructed to listen as many times as needed until they were satisfied with their codes, but a minimum of two complete “listens” was required. Next, each coder provided a single global rating of each domain. Ratings ranged from a score of 1 (not at all present) to 5 (very much present). Discrepancies between coders were resolved via review of notes coders took while listening to interviews to assist with their coding. A copy of the coding manual is included in Appendix 2.

#### Data Analysis

All data analysis was conducted using R (R Core Team, 2016). For variables included in the data analysis, missing data ranged from 0 to 4.8%. Missing data were handled using multiple imputation. Before conducting analyses of the main hypotheses, total utilization scores, WHODAS, and IDS-SR scores were assessed for normality. A Kolmogorov–Smirnov test indicated that data were normally distributed and thus that linear modeling would be an appropriate method of approach. An alpha level of 0.05 was used throughout.

For the first aim, Cronbach’s α was calculated to examine internal consistency, using 0.70 as the recommended cutoff (Cortina, 1993). ICC was calculated using the original ratings made by coders for Overall Engagement and Total Domains prior to resolution of differing ratings. Weighted kappa was used to determine interrater reliability for individual domains, which were evaluated on a 1–5 Likert scale. These scores were interpreted based on cutoffs provided from previous research (Cicchetti & Allison, 1971; Cohen, 1968). For this study, Total Domains was operationalized as the total number of domains scored higher than 1 for a given participant.

For the second aim*,* means and standard deviations were calculated for individual domains, Overall Engagement and Total Domains and are presented alongside Cohen’s *d* (Cohen, 2013) as measures of effect size. Additionally, multiple regression was conducted to examine the extent to which CT + MS compared to CT-as-usual and cumulative recall post-treatment were related to (a) a greater engagement with the 14 domains (Overall Engagement), (b) a higher total number of the 14 domains scored as present (Total Domains), and (c) higher engagement on individual domains. These analyses included years of education at baseline and the number of CT sessions received over the course of participation in the main study (range 20–26 sessions) as covariates.

For the third aim, regression analyses were conducted to examine the relation between Overall Engagement, Total Domains, and individual domains and (a) treatment utilization, (b) WHODAS scores, and (c) IDS-SR scores at 12-month follow-up. For these analyses, treatment condition was included as a covariate, along with years of education at baseline and the number of CT sessions received over the course of participation in the main study (range: 20–26 sessions).

Following prior research (Gumport et al., 2019), we elected to examine only the relations between individual behavior change domains and relevant variables that were significantly associated with Overall Engagement or Total Domains, in order to reduce multiple comparisons. This procedure minimizes the need for overly restrictive alpha corrections.

## Results

A subset of participants from the parent trial received the Behavior Change Interview and were included in data analysis (*n* = 64; age *mean* [SD] = *37.97* [15.77]; 62.5% female), 35 of whom received CT + MS and 29 of whom received CT-as-usual. Demographic information is provided in Supplemental Materials (Supplemental Table S1). Chi-square and t-test analysis revealed that female participants were more likely to have received memory support. There were no other significant differences between the CT + MS and CT-as-usual groups.

### Internal Consistency and Interrater Reliability of the Cognitive Behavior Change Interview (Aim 1)

Internal consistency for the combination of individual domains was acceptable (Cronbach’s α = 0.82), surpassing the acceptable threshold of 0.70 (Cortina, 1993). Interrater reliability was assessed for Overall Engagement and Total Domains, which yielded scores in the excellent and good ranges, respectively (ICCs = 0.79 for Overall Engagement and 0.74 Total Domains; *p* values < 0.001) (Cicchetti & Allison, 1971). Weighted kappa values for all domains except beliefs about capabilities, intentions, and goals, which were measured quantitatively by self-report, are displayed in Supplemental Materials (Supplemental Table S2). Interrater reliability scores for individual domains ranged from unreliable to excellent (Cohen, 1968). All domains were included in subsequent analyses.

### The Cognitive Behavior Change Interview, MSI, and Cumulative Recall (Aim 2)

#### The impact of TREATMENT condition on Overall Engagement, Total Domains, and Individual Domains

Averages for Overall Engagement, Total Domains, and individual domains at the 12-month follow-up are presented in Table 3 alongside descriptive statistics for all relevant variables. For all individual domains except for Reinforcement, Behavior Regulation, and Emotion, average scores were higher for participants who received CT + MS although not all changes were significant. The domains with the largest effect sizes, were Knowledge (*d* = *0.39*), Beliefs about Capabilities (*d* = 0.56), and Beliefs about Consequences (*d* = 0.49). Medium effects were found for Overall Engagement (*d* = 0.57).

**Table 3:** Descriptive statistics of relevant variables by treatment condition (CT + MS vs. CT-as-Usual)

Variable	CT + MS (n = 35)		CT-as-usual (n = 29)		Cohen's *d*
	Mean	SD	Mean	SD	
*Cognitive behavior change interview*					
Knowledge	3.34	0.84	3.03	0.77	0.39
Skills	2.59	0.87	2.55	0.99	0.04
Social role and professional identity	1.80	0.99	1.59	1.02	0.22
Beliefs about capabilities	74.66	15.81	62.62	27.13	0.56
Optimism	3.49	0.80	3.33	0.82	0.20
Beliefs about consequences	3.23	0.97	2.76	0.96	0.49
Reinforcement	1.67	0.69	1.67	0.67	0.00
Intentions	89.14	15.69	85.24	22.65	0.21
Goals	84.57	17.67	77.21	24.42	0.36
Memory, attention, and decision making	2.77	0.74	2.50	0.95	0.33
Environmental context and skills	1.80	0.86	1.64	0.78	0.20
Social Influences	2.17	1.03	1.95	0.97	0.23
Emotions	2.50	1.32	2.53	1.36	− 0.01
Behavioral regulation	2.44	1.03	2.45	0.84	− 0.03
Total domains	11.00	1.49	10.97	2.44	0.01
Overall engagement	2.87	0.45	2.57	0.62	0.57
*Memory support intervention*					
Cumulative recall	12.71	4.68	13.31	5.45	− 0.12
Constructive memory support	4.95	2.16	2.63	1.26	1.30
Non-constructive memory support	11.23	3.95	5.63	1.86	1.79
*Treatment outcomes*					
Treatment utilization	2.44	0.66	2.20	0.74	0.34
WHODAS	20.66	14.46	23.93	17.60	− 0.21
IDS-SR	18.43	10.33	21.93	11.50	− 0.33
*Covariates*					
Years of education	15.49	5.97	16.86	3.60	− 0.28
Number of sessions	23.43	2.17	22.31	3.81	0.38

Beliefs about Capabilities, Intentions and Goals were rated on a scale form 0–100 via self-report

As evident in Table 4, relative to CT-as-usual, CT + MS was significantly associated with higher Overall Engagement (Beta = 0.66, *p* = 0.039). CT + MS was not significantly associated with Total Domains.

**Table 4:** Impact of treatment (CT + MS vs. CT-as-usual) and patient memory for treatment (cumulative recall post-treatment on total domains and overall engagement

Variable		Beta	SE	*P* value
Outcome	Predictor			
Overall engagement	Treatment	0.66	0.31	0.039*
	Cumulative Recall	0.05	0.02	0.029*
Total domains	Treatment	0.47	0.32	0.144
	Cumulative Recall	0.04	0.02	0.066

*Beta* = Standardized beta estimate. ***p <.001, **p < 0.01, *p < 0.05

Because CT + MS was found to have a significant effect on Overall Engagement, exploratory analyses were conducted to examine the effect of CT + MS on individual domain scores, which are presented in Table 5. CT + MS was significantly associated with higher scores in Beliefs about Consequences (Beta = 0.79, *p* = 0.018), but no other domains.

**Table 5:** Impact of treatment (CT + MS vs. CT-as-usual) and cumulative recall at post-treatment on individual domains

Domain	Beta	SE	p-value
*Treatment (CT + MS vs. CT-as-usual)*			
Knowledge	0.29	0.31	0.347
Skills	0.40	0.32	0.220
Social role and professional identity	− 0.05	0.34	0.883
Beliefs about capabilities	0.53	0.32	0.107
Optimism	0.41	0.33	0.217
Beliefs about consequences	0.79	0.33	0.018*
Reinforcement	− 0.06	0.34	0.871
Intentions	0.20	0.30	0.516
Goals	0.23	0.35	0.509
Memory, attention and decision making	0.56	0.33	0.091
Environmental context and skills	0.25	0.34	0.459
Social influences	0.25	0.35	0.482
Emotions	0.31	0.34	0.366
Behavioral regulation	0.09	0.33	0.781
*Cumulative recall at post-treatment*			
Knowledge	0.08	0.02	< 0.001***
Skills	0.07	0.02	0.004**
Social role and professional identity	0.04	0.03	0.157
Beliefs about capabilities	0.02	0.02	0.507
Optimism	0.01	0.02	0.783
Beliefs about consequences	0.04	0.02	0.142
Reinforcement	0.02	0.03	0.442
Intentions	− 0.01	0.02	0.795
Goals	0.02	0.03	0.568
Memory, attention and decision making	0.04	0.02	0.122
Environmental context and skills	0.02	0.03	0.417
Social influences	0.01	0.03	0.606
Emotions	0.06	0.03	0.014*
Behavioral regulation	0.01	0.02	0.605

*Beta* = Standardized beta estimate. ***p <.001, **p < 0.01, *p < 0.05

#### The Impact of Patient Memory for Treatment on Overall Engagement, Total Domains, and Individual Domain Scores

As displayed in Table 4, cumulative recall at post-treatment was significantly associated with Overall Engagement at 12-month follow-up, such that improved cumulative recall predicted higher Overall Engagement (Beta = 0.05, *p* = 0.029). Cumulative recall at post-treatment was not significantly associated with Total Domains at 12-month follow-up.

Because cumulative recall was found to have a significant relation with Overall Engagement, exploratory analyses were conducted to examine the relation between cumulative recall and individual domains. As evident in Table 5, higher cumulative recall post-treatment significantly predicted higher scores in Knowledge (Beta = 0.08, *p* < 0.001), Skills (Beta = 0.07, *p* = 0.004), and Emotions (Beta = 0.06, *p* = 0.014) at 12-month follow-up.

### The Cognitive Behavior Change Interview and Select Treatment Outcomes (Aim 3)

#### The Impact of Overall Engagement, Total Domains, and Individual Domains on Treatment Utilization

As shown in Table 6, Overall Engagement and Total Domains were both significantly associated with Treatment Utilization, such that higher scores in Overall Engagement (B = 0.86, *p* < 0.001) and Total Domains (B = 0.16, *p* = 0.014) were related to greater use of skills learned in treatment at 12-month follow-up.

**Table 6:** Impact of total domains and overall engagement on treatment utilization, disability scores and depression scores

Variables		Beta	SE	*P* value
Outcome	Predictor			
Treatment utilization	Overall engagement	0.86	0.22	< 0.001***
	Total domains	0.16	0.07	0.014*
WHODAS	Overall engagement	− 0.89	0.22	< 0.001***
	Total domains	− 0.21	0.06	0.002**
IDS-SR	Overall engagement	− 0.71	0.23	0.003**
	Total domains	− 0.13	0.07	0.055

*Beta* = Standardized beta estimate. ***p <.001, **p < 0.01, *p < 0.05

Further exploratory analysis of individual domains as predictors of Treatment Utilization are displayed in Table 7. Higher scores in Beliefs about Capabilities (B = 0.03, *p* = 0.014), Optimism (Beta = 0.62, *p* < 0.001), Intentions (Beta = 0.03, *p* < 0.001), and Memory, Attention and Decision Making (Beta = 0.52, *p* = 0.001) were related to greater Treatment Utilization.

**Table 7:** Impact of individual domains on total treatment utilization, disability (WHODAS), depression (IDS-SR) at the 12-month follow-up

Domain	Beta	SE	p-value
*Total treatment utilization*			
Knowledge	0.10	0.16	0.526
Skills	− 0.03	0.14	0.821
Social role and professional identity	0.16	0.13	0.205
Beliefs about capabilities	0.03	0.01	< 0.001***
Optimism	0.62	0.15	< 0.001***
Beliefs about consequences	0.27	0.13	0.042
Reinforcement	0.24	0.19	0.214
Intentions	0.03	0.01	< 0.001***
Goals	0.01	0.01	0.133
Memory, attention and decision making	0.52	0.14	0.001**
Environmental context and skills	− 0.11	0.16	0.509
Social influences	− 0.05	0.13	0.705
Emotions	0.14	0.09	0.147
Behavioral regulation	0.23	0.14	0.111
*WHODAS*			
Knowledge	− 0.16	0.16	0.330
Skills	− 0.29	0.14	0.037*
Social role and professional identity	− 0.28	0.13	0.031*
Beliefs about capabilities	− 0.03	0.01	< 0.001***
Optimism	− 0.44	0.16	0.007**
Beliefs about consequences	− 0.16	0.13	0.223
Reinforcement	− 0.34	0.19	0.077
Intentions	− 0.02	0.01	0.018*
Goals	− 0.02	0.01	0.011*
Memory, attention and decision making	− 0.26	0.16	0.100
Environmental context and skills	− 0.10	0.16	0.525
Social influences	0.05	0.13	0.729
Emotions	− 0.25	0.09	0.008**
Behavioral regulation	− 0.26	0.14	0.069
*IDS-SR*			
Knowledge	0.23	0.16	0.146
Skills	− 0.11	0.14	0.441
Social role and professional identity	− 0.21	0.13	0.101
Beliefs about capabilities	− 0.03	0.01	< 0.001***
Optimism	− 0.74	0.14	< 0.001***
Beliefs about consequences	− 0.22	0.13	0.105
Reinforcement	− 0.12	0.19	0.535
Intentions	− 0.02	0.01	0.022*
Goals	− 0.02	0.01	0.010*
Memory, attention and decision making	− 0.25	0.16	0.122
Environmental context and skills	0.08	0.16	0.613
Social influences	0.18	0.13	0.155
Emotions	− 0.17	0.09	0.082
Behavioral regulation	− 0.16	0.14	0.270

*Beta* = Standardized beta estimate. ***p <.001, **p < 0.01, *p < 0.05

#### The Impact of Overall Engagement, Total Domains, and Individual Domains on Impairment

Overall Engagement and Total Domains were significantly associated with WHODAS scores at 12-month follow-up, such that higher Overall Engagement (Beta = − 0.89, *p* < 0.001) and Total Domains (Beta = − 0.21, *p* = 0.002) were related to lower scores on the WHODAS, indicating lower levels of impairment.

Exploratory analysis of individual domains as predictors of WHODAS scores is presented in Table 7. Skills (Beta = − 0.29, *p* = 0.037), Social Role and Professional Responsibility (Beta = − 0.28, *p* = 0.031), Beliefs about Capabilities (Beta = − 0.03, *p* < 0.001), Optimism (Beta = − 0.44, *p* = 0.007), Intentions (Beta = − 0.02, *p* = 0.018), Goals (Beta = − 0.02, *p* = 0.011), and Emotions (Beta = − 0.25, *p* = 0.008) were significantly related to WHODAS scores.

#### The Impact of Overall Engagement, Total Domains, and Individual Domains on Depression

Overall Engagement was significantly associated with IDS-SR scores at 12-month follow-up, such that higher Overall Engagement was related to lower scores on the IDS-SR (Beta = − 0.13, *p* = 0.003). Total Domains was not significantly associated with IDS-SR scores.

Exploratory analysis on individual domains as predictors of IDS-SR scores are displayed in Table 7. Beliefs about Capabilities (Beta = − 0.03, *p* < 0.001), Optimism (Beta = − 0.74, *p* < 0.001), Intentions (Beta = − 0.02, *p* = 0.022), and Goals (Beta = − 0.02, *p* = 0.010) were significantly related to IDS-SR scores.

## Discussion

The overall goal of this study was to better understand the potential role of memory support and patient memory for treatment in improving long-term behavior change among recipients of cognitive therapy for depression. Additionally, this study sought to evaluate the Cognitive Behavior Change Interview as a measure to improve understanding of behavior change in the context of the MSI. At the outset, it is important to note that the two groups differed on gender. This will be addressed in the limitations section below.

The first aim was to establish the internal consistency and interrater reliability of the Cognitive Behavior Change Interview. There was strong evidence for internal consistency across domains for the Cognitive Behavior Change Interview. This finding suggests that individual domain scores are related to one another, and that overall engagement, which reflects the extent to which all domains in the TDF are present for a given participant, can be considered a single construct. The finding that domains may be related to one another is novel, as relations across domains are not addressed within the TDF (Atkins et al., 2017). Future research exploring relations and potential interactions across domains as they relate to outcomes in the context of the MSI is recommended.

The analyses for interrater reliability yielded mixed results. Overall Engagement and Total Domains had strong interrater reliability. Contrasted with Overall Engagement, Total Domains reflects the total overall number of domains that are present for a given participant. When examining individual domains, interrater reliability was strongest for Social Influences, Social Role and Professional Identity, and Optimism. However, the kappa estimate for Skills indicated poor interrater reliability. This finding raises the possibility that coders might have benefited from additional training in cognitive therapy, allowing them to more consistently identify therapy skills that clients used even when not explicitly labelled. Increased knowledge in the core concepts of cognitive behavioral therapy might have also improved the kappa estimates for environmental context and skills, as well as beliefs about consequences, which also yielded low kappa estimates. Alternatively, it is possible that the range of skills sampled was sufficiently diverse such that consistent coding was not possible. There may also be a benefit to pre-selecting domains that are likely to be influenced by cognitive therapy and memory support according to preexisting research, reducing the number of domains in which coders would need training.

The second aim was to use the Cognitive Behavior Change Interview to better understand the MSI and patient memory for treatment. We first focused on establishing whether patients who received the MSI scored better on Overall Engagement, Total Domains, or individual domain scores. In partial support of this hypothesis, receiving CT + MS vs. CT-as-usual positively predicted Overall Engagement and better patient memory for treatment also positively predicted Overall Engagement. However, neither receiving CT + MS nor improving patient memory for treatment was related to Total Domains. Taken together, these findings indicate that CT + MS may function to improve how CT engages with select domains of the TDF but does not utilize more domains from the TDF overall when compared to CT-as-usual.

Due to the kappa estimates, analyses of individual domains is considered exploratory. However, these exploratory analyses suggested that receiving CT + MS predicted Beliefs about Consequences, while better patient memory for treatment predicted better Knowledge, Skills, and Emotion scores at the 12-month follow-up. These findings suggest that participants who received CT + MS have a better understanding of how addressing negative thoughts via skills might influence their mood relative to CT-as-usual. This finding aligns with specific forms of memory support provided by the MSI, such as “Application,” which asks participants to consider how using a skill learned in therapy may help them personally.

We next explored whether patients who exhibit better memory for treatment at the post-treatment assessment also score higher on Overall Engagement, Total Domains, and individual domains at the 12-month follow-up. Although patient memory for treatment was not found to be related to Total Domains, better patient memory for treatment was associated with higher Overall Engagement as well as higher scores in Knowledge, Skills, and Emotion domains. Perhaps memory for treatment is associated with Knowledge and Skills because these domains might fundamentally build toward better patient memory for treatment. Also, it is possible that CT + MS may assist with improving beliefs about consequences because understanding the consequences of skill use requires participants to actively apply treatment contents to their own lives, which is a more complicated task relative to the free recall performed during the Patient Recall Task (Zieve et al., 2022). Taken together, these findings are consistent with previous research indicating that the MSI improves patient learning for treatment (Dong et al., 2022). Furthermore, the association between patient memory for treatment and information-based domains, such as knowledge and skills, could also be used as preliminary evidence of construct validity for the Cognitive Behavior Change Interview.

The third aim was to examine the relation between Overall Engagement, Total Domains, and three treatment outcomes: treatment utilization, disability, and depression. Consistent with hypotheses, Overall Engagement and Total Domains predicted treatment utilization. These findings indicate that higher scores on these variables were related to more frequent use of treatment elements by participants 12-months after the end of treatment. In other words, higher scores on Overall Engagement were related to measurable differences in later behavior. This finding serves as validation for the TDF, which was created to organize common components of behavior change according to previous research and theory under one framework (Cane et al., 2012; Horppu et al., 2017). The results are also consistent with the broader literature on behavior change, which established the domains listed in the TDF as predictors of behavior (Cane et al., 2012; Sheeran et al., 2016).

The exploratory analyses conducted on individual domains indicated that higher scores in Beliefs about Capabilities, Optimisms, Intentions and Memory, Attention and Decision Making significantly predicted higher treatment utilization at 12-month follow-up. This pattern of findings is consistent with the broader literature highlighting the role of constructs such as self-efficacy—a component of beliefs about capabilities as defined by Cane et al. (2012)—optimism, intention, and prospective memory in behavior change (Baldwin et al., 2006; Bandura, 1998; Sheeran et al., 2016). The Theory of Planned Behavior, one of the most applied theories across social and behavioral sciences (Bosnjak et al., 2020), emphasizes the role that intention and self-efficacy (particularly perceived behavioral control) play in behavior change (Ajzen, 1991; Bosnjak et al., 2020), thus linking Beliefs about Capabilities to behavior change. It also includes a role for attitude, which can be extended to optimism (Ajzen, 1991).

In terms of disability and impairment, Overall Engagement and Total Domains predicted WHODAS scores. These findings suggest that higher Overall Engagement and Total Domains are associated with improved outcomes for patients. Given the connection between TDF domains and treatment outcomes, behavior change appears to be one of the many paths through which memory support can enhance treatment outcomes. In terms of the exploratory analyses, Skills, Social Role and Professional Responsibility, Beliefs about Capabilities, Optimism, Intentions, Goals and Emotions were significantly related to WHODAS scores. Several of the individual domains were negatively associated with disability. It is possible that participants who have a better understanding of skills and beliefs about capabilities may be more likely to utilize coping mechanisms that improve functioning (Schwarzer et al., 2011). As highlighted by Michie’s behavior change wheel, participants who are more optimistic and intend to use skills learned in treatment may be more motivated to take advantage of skills learned in treatment and also resources within their environment that reduce disability (Michie et al., 2011).

In terms of depression, patient beliefs about Capabilities, Optimism, Intentions, and Goals were also related to lower depression scores. Extensive research has been conducted on depression and goal-setting, providing evidence that a goal-focused intervention can improve well-being in adults with depression (Coote & MacLeod, 2012). On the other hand, the Cognitive Behavior Change Interview and IDS-SR were administered at the same timepoint. Hence, it is important to consider that participants who are less depressed may be more optimistic, have goals for the future, and feel more motivated to use therapy skills.

This research has several limitations. First, the Cognitive Behavior Change Interview was administered only at 12-month follow-up on a subset of participants from the original study. Collecting data on TDF domains at this time provided an examination of the long-term effects of CT + MS. However, by assessing only the 12-month follow up, we were unable to establish the directionality of any relations between domains and outcomes. Also, administering the Cognitive Behavior Change Interview at an earlier timepoint would have provided additional insight regarding the test–retest reliability of the measure. Second, although internal consistency was high, as was the interrater reliability for composite scores, kappa scores measuring the interrater reliability of select individual domains was weak. However, analyses of individual domains were exploratory in nature, and interrater reliability for Overall Engagement and Total Domains was acceptable. Finally, female participants were more likely to have received memory support. The possible impact of this sex difference on the results is not known, and further exploration of the impact of gender on cognitive therapy and memory support outcomes is a topic for future research. It is also worth noting that this research study involved a highly educated sample. It is possible that the MSI may differently impact individuals with lower levels of education who may have less practice memorizing and being tested on facts.

In summary, the results from the present study provide preliminary evidence that improving patient memory for treatment via the MSI facilitates behavioral changes beyond what was provided by CT alone. This research highlights individual domains that may play an important role in treatment outcomes and long-term behavior change, such as Beliefs about Capabilities and Knowledge which may increase self-efficacy. These findings expand our understanding of the MSI and contribute to the expanding body of research that uses the TDF to improve our understanding of evidence-based interventions and their results.

## Supplementary Information

Below is the link to the electronic supplementary material.

Supplementary file1 (DOCX 169 kb)

## Data Availability

Data are available from the authors upon reasonable request.
