# Supplementary material for: Do the Memory Support Intervention and Improving Memory for Treatment Facilitate Behavior Change in Cognitive Therapy?
Source: Cognit Ther Res. 2025 Jun 18;50(1):1–13. doi: 10.1007/s10608-025-10629-7 (PMC13002768; doi:10.1007/s10608-025-10629-7)
Supplement: Supplementary file 1 — Supplementary file1 (DOCX 169 kb) [file 10608_2025_10629_MOESM1_ESM.docx]

**Supplemental Materials**

1. Cognitive Behavior Change Interview
2. Coding Manual
3. Supplemental Table S1: Sample Characteristics of Patients in CT+MS versus CT-as-usual
4. Supplemental Table S2: Weighted Kappa Estimates for Individual Behavior Change Domains

**1. Cognitive Behavior Change Interview**

| Participant ID: | Interviewer: |
| --- | --- |
| Date: | Participant’s Last Session of CBT: |
| Most important skill(s): |  |

**Cognitive Behavior Change Interview Introduction**

Thank you so much for taking the time to speak with me today. I appreciate your willingness to tell us a little bit more about your experience with CBT and what you learned.

**Capability**

**Knowledge**

1. What did you learn in therapy about managing negative thoughts? (Prompt: what do you know about why we should manage negative thoughts)

**Skills**

1. Interview Question: What skills help you manage your negative thoughts? (Prompt: Which skill has been most useful to you? Anything else?) *Note: At this point, the interviewer should take note of specific CBT skills the client uses to manage their thoughts, and write the most important skill at the top of the page*

**Memory, Attention and Decision Processes**

1. When you have negative thoughts, what helps you remember to use the skills you learned in CBT? For example, [name the most important skill that was listed earlier]. (Prompt: Do you feel like it's easy to remember to use skills when you're "in the moment"?)
2. How do you decide which skills to use when you have negative thoughts? What influences your decision? (Prompt: Does it happen automatically? How hard is it to choose a skill?)

**Behavioral Regulation**

1. What do you do to make sure that you continue practicing [your most important skill] over time?

**Opportunity**

**Environment**

1. Is there anything about your environment that makes it easier to [use your most important skill]? (Prompt: If the participant cannot think of any, provide the following example: “For example, some people set reminders in their phones, leave their Mind Over Mood book in a visible place, or have specific times and places where it’s easiest to use skills)

**Social Influence**

1. Are there people in your life who help you [use your most important skill] or make it easier to manage negative thoughts? (Prompt: Who? How?)

**Motivations**

**Social Role/Identity**

1. Are there parts of your job or identity that make it easier to [use your most important skill]?

**Optimism**

1. Are you confident that negative thoughts can be managed?
2. Do you think that if people manage their negative thoughts over time, it can lead to improved mood?

**Beliefs about Capabilities (Self-Efficacy)**

1. On a scale from 0-100, how confident are you in your ability to use skills to manage your negative thoughts (100 would be very confident)? Why?
2. How did your therapist ensure that you had the tools to manage negative thoughts? (Prompt: Tell me more about that)

**Beliefs about Consequences**

1. How do you feel after [practicing your most important skill]? Can you tell me about a time that you used it? (Prompt: Was that successful? How does using the skills you learn affect you personally?)

**Reinforcement**

1. Have you had any experiences that have influenced whether or not you [use your most important skill]? (Prompt: was that built into therapy at all? How did you and your therapist talk about these experiences?)

**Goals**

1. On a scale from 0-100, how important is it to you that you manage your negative thoughts? (Prompt: Why?)

**Intentions**

1. On a scale from 0-100, how committed are you to using the skills you learned to manage your negative thoughts?

**Emotions**

1. Do your emotions ever affect your ability to manage your thoughts with skills? What's that like? How do you ensure that you use your skills anyway? (Prompt: Which emotions? Do they help or make things harder?)

**2. Coding Manual**

**Domain Definitions and Scoring Guide:**

The domains below use the definitions and constructs defined in Cane et al (2012).

Each domain should be scored on a scale from 1-5 below, giving a macro-level impression of each domain as it relates to the management of negative automatic thoughts (NATs).

**Significance of Each Rating**

1. Not at all present
2. Barely present/low quality
3. Somewhat present/moderate quality
4. Quite present/ high quality
5. Very much present/ Very high quality

Below, you will find further information concerning the contextual relevance of each domain to NATs. The scoring section should provide guidance regarding the presence of a specific domain.

**Knowledge**

*Definition:*An awareness of the existence of something.

*Constructs*: Knowledge, procedural knowledge, knowledge of task environment

*Scoring:* In the context of cognitive therapy, a participant should understand what negative automatic thoughts are and why cognitive therapy focuses on thoughts. Information the participant provides about the cognitive model or personal situations that may trigger NATs should also be included in the scoring. This can include defining what negative automatic thoughts are, how to catch negative automatic thoughts, why NATs are important, situations that are likely to trigger NATs for the individual participant, and how examples of how NATs influence mood and behavior.

**Skills**

*Definition:*An ability or proficiency acquired through practice

*Constructs*: Skills, skills development, competence, ability, interpersonal skills, practice, skill assessment

*Scoring:*  When scoring “skills,” coders should count the distinct number of skills participants recall for the purpose of managing negative automatic thoughts. Coders should also look for instances in which a participant can explain how a skill works without naming the skill explicitly, alludes to a level of proficiency with that skill, or describes practicing the skill both during and outside therapy.

**Social/Professional Role and Identity**

*Definition:*A coherent set of behaviors and displayed personal qualities of an individual in a social or work setting

*Constructs:*Professional Identity, professional role, social identity, identity, professional boundaries, professional confidence, group identity, leadership, organizational commitment

*Scoring:* In the context of CT, coders should listen for indications that the skills learned in CT align with a participant’s personal values and professional duties or expectations. For example, do they believe that it’s part of their job to learn certain skills or that the use of specific skills is consistent with their workplace responsibilities?

**Beliefs about Capabilities**

*Definition:*Acceptance of the truth, reality, or validity about an ability, talent, or
facility that a person can put to constructive use

*Constructs:*Self-confidence, perceived competence, self-efficacy, perceived behavioral control, beliefs, self-esteem, empowerment, professional confidence

*Scoring:* This domain is scored by the participant, who rates their ability to manage negative thoughts.

**Optimism**

*Definition*: The confidence that things will happen for the best or that desired goals will be attained

*Constructs:*Optimism, pessimism, unrealistic optimism, identity

*Scoring:* Coders should take note of whether or not a participant believes negative thoughts can be controlled more generally. Furthermore, statements about a participant’s perceived ability to continue improving their skills over time and achieve their goals should be factored into scoring.

**Beliefs about Consequences**

*Definition:* Acceptance of the truth, reality, or validity about outcomes of a
behavior in a given situation

*Constructs:*Beliefs, outcome expectancies, characteristics of outcome expectancies, anticipated regret, consequents

*Scoring:* Coders should look for whether or not participants can recall the consequences of using skills both generally and in specific examples. Additionally, look for whether or not participants can compare cases when skills are used to cases when they are not. Note any general advantages or disadvantages of using skills that are discussed.

**Reinforcement**

*Definition:*Increasing the probability of a response by arranging a dependent relationship, or contingency, between the response and a given stimulus

*Constructs:*Rewards, incentives, punishment, consequences, reinforcement, contingencies, sanctions.

*Scoring: Note* anything that reinforces skill use. Two additional points should be awarded in instances where a participant has intentionally arranged a contingency where a reward is provided for skill use.

**Intentions:**

*Definition: ﻿*A conscious decision to perform a behavior or a resolve to act in a certain way.

Constructs: Stability of intentions, stages of change, transtheoretical model and stages of change

*Scoring:* This domain is scored by the participant, who rates their commitment to managing thoughts.

**Goals:**

*Definition:* Mental Representations of outcomes or end states that an individual wants to achieve

Constructs: ﻿Goals (distal/proximal), goal priority, goal/target setting; goals (autonomous/controlled), action planning, implementation intention

*Scoring:* This domain is scored by the participant, who rates the importance of managing negative automatic thoughts as a goal.

**Memory Attention and Decision Making:**

*Definition:* The ability to retain information, focus selectively on aspects of the environment, and choose between two or more alternatives.

Constructs: Memory, attention, attention control, decision making, cognitive overload/tiredness

*Scoring:* Coders should look for techniques, cues or routines that help participants remember to use skills and note any comments on the effectiveness of those techniques or routines. Coders should also note any description of decision-making processes that help a participant choose which skill to use. Answers such as going to the first score you remember, or your favorite skill, do not count.

**Environmental Context and Skills:**

*Definition:* ﻿Any circumstance of a person's situation or environment that discourages or encourages the development of skills and abilities, independence, social competence, and adaptive behavior

Constructs: ﻿Environmental stressors, resources/material resources, organizational culture/climate, salient events/critical incidents, person x environment interaction, barriers and facilitators

*Scoring:* Coders should look for physical cues that participants put in place to remind them to practice skills. Specific environments, opportunities or external factors that are used to practice skill use should also factor into the rating of this domain. For example, a participant may use their morning commute as an opportunity to complete a behavioral experiment, or practice gratitude while they garden.

**Social Influences**

**﻿** *Definition:* Those interpersonal processes that can cause individuals to change their thoughts, feelings, or behaviors

Constructs: ﻿Social pressure; Social norms, group conformity, social comparisons, group norms, social support, power, intergroup conflict, alienation, group identity, modeling

*Scoring:* Coders should pay attention to the number of people who help them practice skills, and also social norms that positively influence skill use. Coders should discriminate between people in a participant’s life who explicitly encourage skill use vs. those who are supportive in other ways, such as being someone the participant can go to in order to vent.

**Emotions:**

*Definition:* *﻿*A complex reaction pattern involving experiential, behavioral, and physiological elements by which the individual attempts to deal with a personally significant matter or event

Constructs: ﻿Fear, anxiety, affect, stress, depression positive, negative affect, burn-out

*Scoring:* Coders should assess the participant’s ability to use skills and manage negative thoughts when they are feeling strong negative emotions. Skills or techniques a participant uses to successfully to recover from emotions should also factor positively into the coder’s rating.

**Behavioral Regulation**

**﻿** *Definition:* Anything aimed at managing or changing objectively observed or measured actions

**﻿** Constructs: Self-monitoring, breaking habit, action planning

*Scoring:* Coders should look for what the participant does to keep track of their skill use. For example, self-monitoring, action planning or habit building. Additionally, coders should look for examples of cue-based reminders and application, such as proactively recognizing that they are in a situation that routinely triggers the same thought and recognizing what skill should be used to manage that.

**3. Supplemental Table S1**

*Sample Characteristics of Patients in CT+MS versus CT-as-usual*

| Characteristic | CT+MS = 35 | | CT-as-usual = 29 | | χ^2^ | p-value |
| --- | --- | --- | --- | --- | --- | --- |
|  | n | % | n | % |  |  |
| Female | 27 | 77.14 | 13 | 44.83 | 5.75 | 0.016 |
| Gender |  |  |  |  | 7.23 | 0.065 |
| Man | 9 | 25.71 | 16 | 55.17 |  |  |
| Woman | 23 | 65.71 | 13 | 44.83 |  |  |
| Preferred not to answer | 3 | 8.57 | 0 | 0.00 |  |  |
| Not specified | 2 | 5.71 | 0 | 0.00 |  |  |
| Ethnicity |  |  |  |  | 0.06 | 0.802 |
| Hispanic or Latino | 3 | 8.57 | 3 | 10.34 |  |  |
| Not Hispanic or Latino | 31 | 88.57 | 25 | 86.21 |  |  |
| Missing | 1 | 2.86 | 1 | 3.45 |  |  |
| Race |  |  |  |  | 4.75 | 0.191 |
| White | 26 | 74.29 | 20 | 68.97 |  |  |
| African American/Black | 1 | 2.86 | 0 | 0.00 |  |  |
| American Indian or Alaskan Native | 0 | 0.00 | 0 | 0.00 |  |  |
| Asian | 7 | 20.00 | 4 | 13.79 |  |  |
| Native Hawaiian/Other Pacific Islander | 0 | 0.00 | 0 | 0.00 |  |  |
| Mixed Race | 1 | 2.86 | 5 | 17.24 |  |  |
| Civil status |  |  |  |  | 6.29 | 0.279 |
| Single | 22 | 62.86 | 15 | 51.72 |  |  |
| Married/common-law partner | 8 | 22.86 | 13 | 44.83 |  |  |
| Separated/divorced/widowed | 5 | 14.29 | 1 | 3.45 |  |  |
| Education |  |  |  |  | 11.81 | 0.160 |
| Some high school | 1 | 2.86 | 0 | 0.00 |  |  |
| Some college | 15 | 42.86 | 5 | 17.24 |  |  |
| Completed college | 4 | 11.43 | 11 | 37.93 |  |  |
| Some graduate school | 0 | 0.00 | 1 | 3.45 |  |  |
| Completed master’s degree | 8 | 22.86 | 8 | 27.59 |  |  |
| Graduate training beyond master's | 3 | 8.57 | 1 | 3.45 |  |  |
| Completed doctorate | 2 | 5.71 | 2 | 6.90 |  |  |
| Missing | 2 | 5.71 | 1 | 3.45 |  |  |
| Employment |  |  |  |  | 8.45 | 0.207 |
| Full-time | 12 | 34.29 | 15 | 51.72 |  |  |
| Part-time | 10 | 28.57 | 5 | 17.24 |  |  |
| Unemployed | 9 | 25.71 | 8 | 27.59 |  |  |
| Other | 4 | 11.43 | 1 | 3.45 |  |  |
| Living arrangement |  |  |  |  | 4.95 | 0.176 |
| Alone | 1 | 2.86 | 4 | 11.43 |  |  |
| With family (spouse, partner, extended family, or children) | 17 | 48.57 | 17 | 48.57 |  |  |
| With friend, roommate, or pet | 16 | 45.71 | 8 | 22.86 |  |  |
| Selected multiple options | 1 | 2.86 | 0 | 0 |  |  |
|  | M | SD | M | SD | t-value | p-value |
| Age (in years) | 37.74 | 17.84 | 38.24 | 13.14 | 0.129 | 0.898 |
| Education (in years) | 15.45 | 6.01 | 16.67 | 3.45 | 0.959 | 0.342 |
| Annual household income (in USD) | 70,464.29 | 67,518.99 | 111,450.00 | 74,837.00 | 1.948 | 0.059 |

Note. M=Means, SD=Standard Deviation. Within this sample, all participants who indicated living with family and a pet were categorized as living with family. Additionally, the “female” marker is indicative of sex assigned at birth and not gender identity.

**4. Supplemental Table S2**

*Weighted Kappa Estimates for Individual Behavior Change Domains*

| Domain | Weighted Kappa |
| --- | --- |
| Knowledge | 0.68 |
| Skills | 0.29 |
| Social Role and Professional Identity | 0.76 |
| Optimism | 0.75 |
| Beliefs about Consequences | 0.53 |
| Reinforcement | 0.58 |
| Memory, Attention, and Decision Making | 0.62 |
| Environmental Context and Skills | 0.43 |
| Social Influences | 0.82 |
| Emotions | 0.58 |
| Behavioral Regulation | 0.42 |

Note. All p-values < 0.05
